# Supplementary figures and images for: Serum heparan sulfate and chondroitin sulfate concentrations in patients with newly diagnosed exfoliative glaucoma
Source: PeerJ. 2019 May 23;7:e6920. doi: 10.7717/peerj.6920 (PMC6535222; doi:10.7717/peerj.6920)

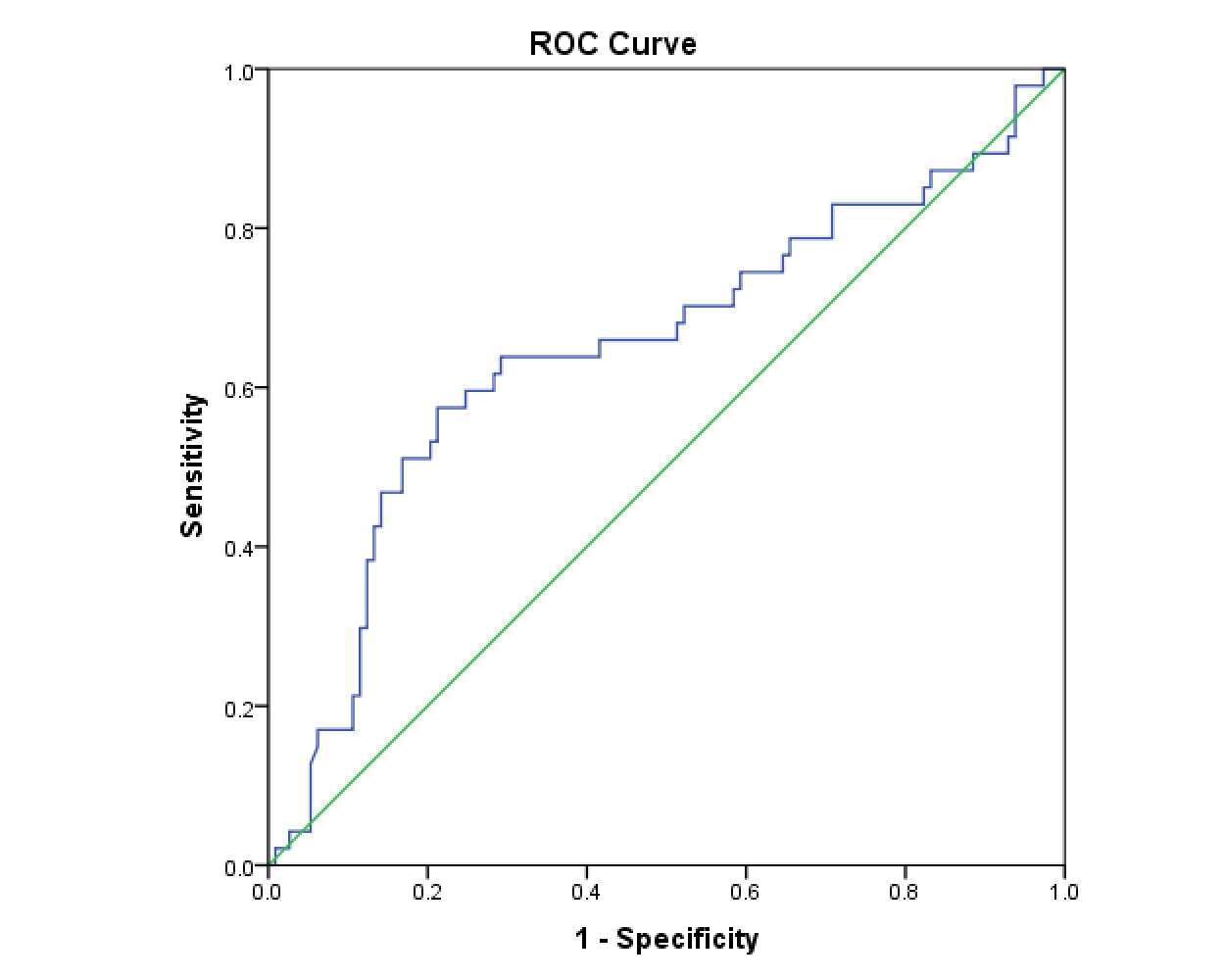

Supplement: Supplemental Information 2 — ROC curve drawn for the comparison of serum HS concentration between patients with XFG and NC, controls with XFS and POAG and AUC was 0.65 (p = 0.003). [file peerj-07-6920-s002.png]

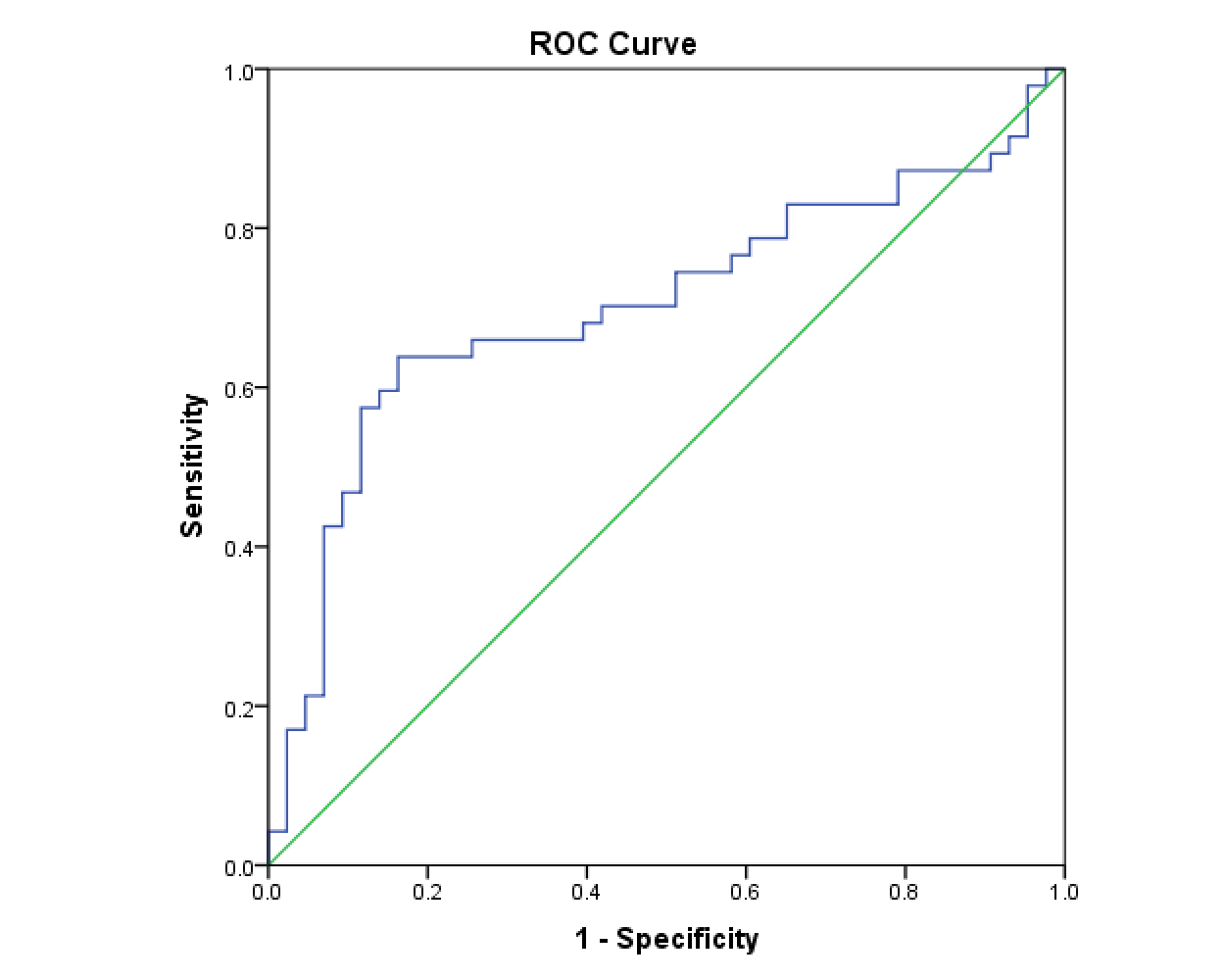

Supplement: Supplemental Information 3 — ROC curve drawn for the comparison of serum HS concentration between patients with XFG and controls with POAG and AUC was 0.70 (95% CI [0.590−0.814]), p = 0.001. [file peerj-07-6920-s003.png]

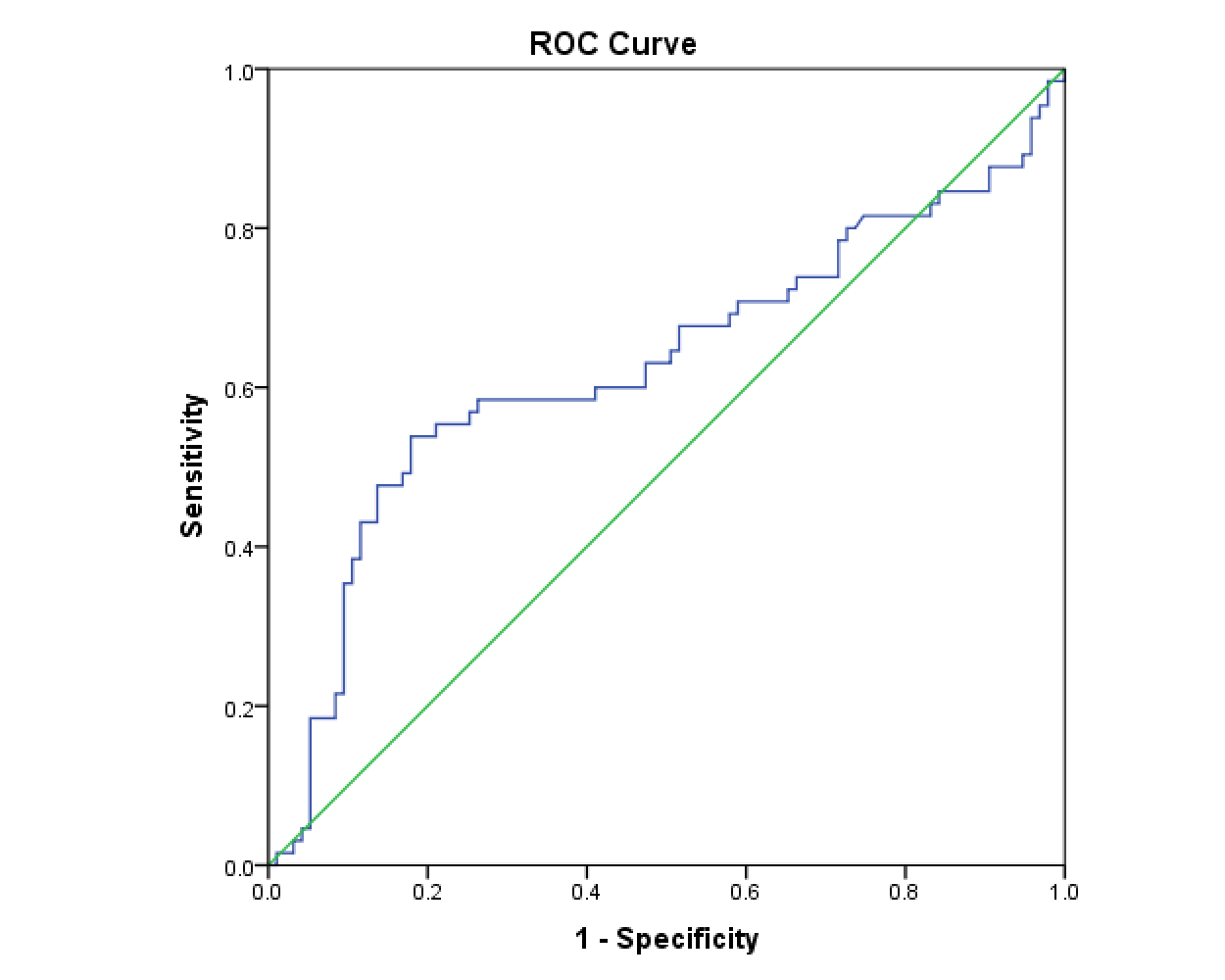

Supplement: Supplemental Information 4 — ROC curve drawn for the comparison of serum HS concentration between subjects with XFM (XFG and XFS) and subjects without XFM (NC and controls with POAG) and AUC was 0.63 (95% CI [0.538−0.721]), p = 0.006. [file peerj-07-6920-s004.png]

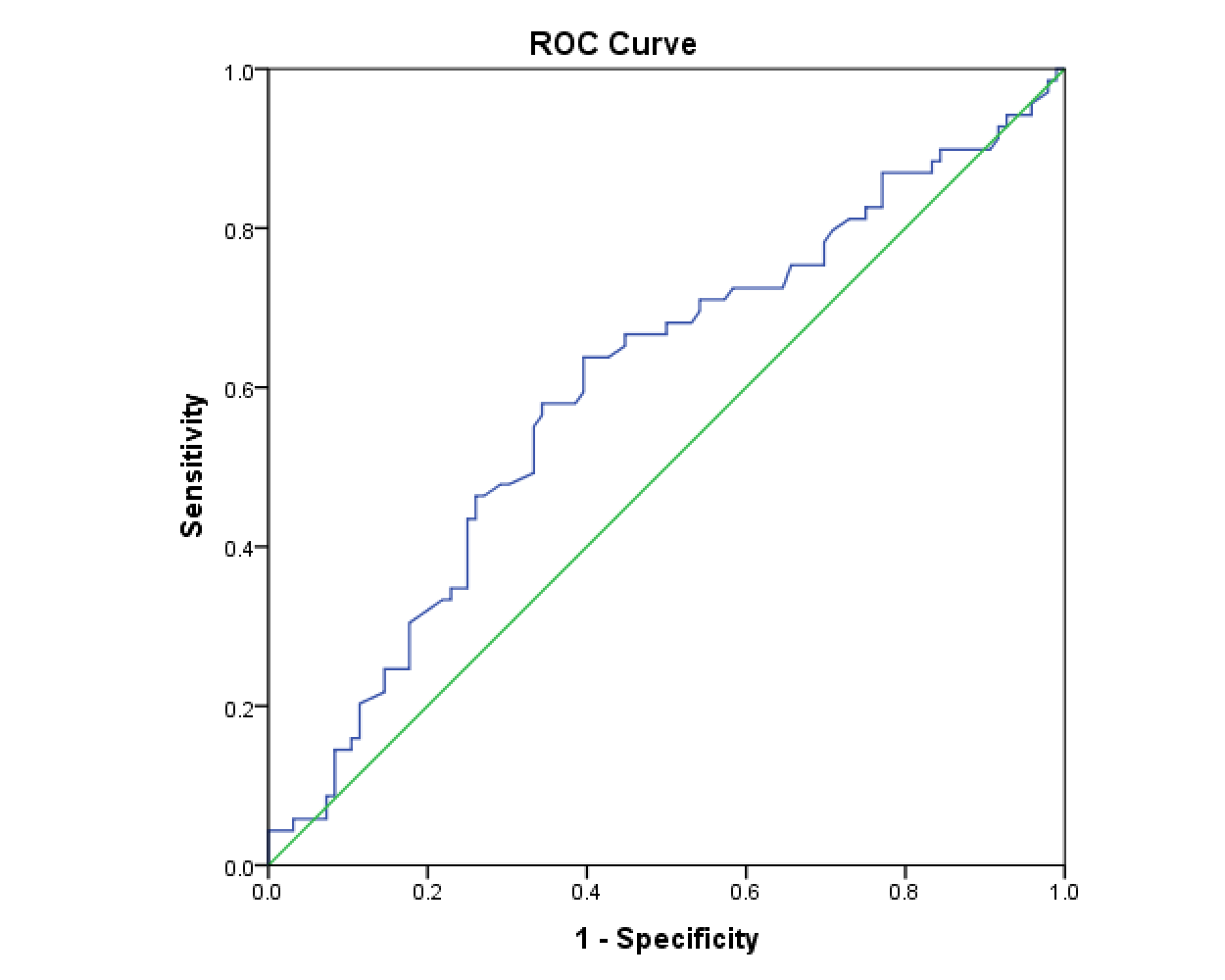

Supplement: Supplemental Information 5 — ROC curve drawn for the comparison of serum CS concentration between subjects with XFM (XFG and XFS) and subjects without XFM (NC and controls with POAG) and AUC was 0.60 (95% CI [0.513−0.690]), p = 0.026. [file peerj-07-6920-s005.png]
